# Integrative Targeted Metabolomics and Transcriptomics Reveal the Mechanism of Leaf Coloration in *Impatiens hawkeri* ‘Sakimp005’

**DOI:** 10.3390/ijms26010174

**Published:** 2024-12-28

**Authors:** Jia-Qi He, Dou-Cheng Yu, Si-Yu Ren, Xiao-Li Zhang, Xin-Yi Li, Mei-Juan Huang, Hai-Quan Huang

**Affiliations:** Research and Development Center of Landscape Plants and Horticulture Flowers, Yunnan Engineering Research Center for Functional Flower Resources and Industrialization, Southwest Research Center for Engineering Technology of Landscape Architecture (State Forestry and Grassland Administration), College of Landscape Architecture and Horticulture Sciences, Southwest Forestry University, Kunming 650224, China; hejiaqi153@swfu.edu.cn (J.-Q.H.); 18184898294@163.com (D.-C.Y.); 17358625583@163.com (S.-Y.R.); m18468178186@163.com (X.-L.Z.); xinyili@swfu.edu.cn (X.-Y.L.)

**Keywords:** leaf color, *Impatiens hawkeri* ‘Sakimp005’, carotenoids, targeted metabolomics, transcriptomics

## Abstract

One of the most important characteristics of ornamental plants is leaf color, which enhances the color of plant landscapes and attracts pollinators for reproduction. The leaves of *Impatiens hawkeri* ‘Sakimp005’ are initially green, then the middle part appears yellow, then gradually become white, while the edge remains green. In the study, leaves of *I. hawkeri* ‘Sakimp005’, in four developmental stages (S1-G, S2-C, S3-C, and S4-C), were selected for the determination of pigment content, chromaticity values, integrative metabolomics, and transcriptomics analyses. The carotenoid content of leaves varied significantly and regularly at four stages, and the colorimetric values corroborated the phenotypic observations. The results of integrative metabolomics and transcriptomics analysis show that the accumulation of two carotenoids (lutein and zeaxanthin), to different degrees in the leaves of *I. hawkeri* ‘Sakimp005’ at four stages, led to the vary yellowing phenomenon. We speculated that the carotenoid biosynthesis (containing two branches: α-branch and β-branch) in leaves by *IhLUT1* and *IhLUT5* in the α-branch and *IhBCH2* genes in the β-branch differed. These findings provide a molecular basis for *Impatiens* plants’ leaf color breeding and improve the knowledge of the leaf color mechanism.

## 1. Introduction

*Impatiens* is an economically important horticultural plant commonly grown around the world for its attractive flowers and medicinal value [1,2]. *Impatiens hawkeri* ‘Sakimp005’ is a well-branched, full-bodied plant with long-lasting salmon pink blooms at the tops of the stems that cover the entire plant. The most distinguishing trait of this species is the leaf color, which starts off pure green, then turns bright yellow in the middle, and finally white yellow, while the edges remain green. Its distinctive leaf color makes it an excellent companion for horticultural settings. It is an excellent genetic resource for developing novel leaf color varieties of the *Impatiens* plant. Currently, most of the studies on *Impatiens* plants focused on flower color [3,4], flower development [5], spur development [6,7], lignin [8], and diseases [9,10], and few studies have been conducted on the coloration of the leaves of *Impatiens* plants, especially on *I. hawkeri* ‘Sakimp005’.

Leaves are the crucial organs for photosynthesis, respiration, and nutrient conversion in plants. They are also the most direct component of plants in the scenery, significantly enhancing the urbanized landscape’s aesthetic appeal [11,12]. A variety of factors contribute to the formation and variation in plant leaf color, including the influence of the external environment, the plant’s internal physiological processes, and the expression of the appropriate genes [13]. For ornamental plants, leaf coloration is an important phenotypic feature that can be easily recognized, giving the most intuitive visual impact, and has long been of interest to breeding experts [14]. A strong theoretical basis for landscape enrichment and crucial theoretical direction for the use and promotion of colorful foliage herbaceous plants will also be established by investigating the physiological and molecular regulatory mechanisms of leaf color change and the associated genes involved in leaf color regulation [15].

Changes in the content of chlorophylls, carotenoids, anthocyanins, and other pigments affect the color of leaves [16]. Carotenoids, the photosynthetic pigments of higher plants, act as co-pigments in chloroplasts and contribute to efficient photosynthesis, protecting plant tissues from excessive light or heat stress and preventing photo-oxidative damage. Carotenoids are found primarily in the leaves, flowers, fruits, roots, and other organs of plants and can appear as red, orange, yellow, or colorless, as well as other colors, and variations in their levels and proportions can significantly affect the color of plant leaves [17,18,19,20]. Research indicated that *Ulmus pumila* contains more amounts of carotenoids, chlorophyll a, and chlorophyll b than *Ulmus pumila* ‘Jinye’, and this difference in carotenoids may have contributed to the yellowing leaves of the *U. pumila* ‘Jinye’ [21]. In a study on the variegated yellow leaves of *Ginkgo biloba*, Li et al. [22] discovered that the yellow leaves had a greater carotenoid concentration than the green leaves, especially the accumulation of lutein, and at the same time, the ratio of the carotenoid to the chlorophyll content increased. The biosynthetic pathway of carotenoids in higher plants has been elucidated [23]: one molecule of dimethylallyl pyrophosphate (DDMAPP) and three molecules of isopentenyl diphosphate (IPP) condense to generate geranylgeranyl pyrophosphate (GGDP), which is the product of the isoprenoid or terpenoid pathways, which are sources of carotenoids. The first colorless carotenoid, octahedron lycopene, is produced by the condensation of two molecules of GGDP. Lycopene is then synthesized by phytoene synthase (*PSY*), phytoene desaturase (*PDS*), 15-cis-ζ-carotene isomerase (*Z-ISO*), ζ-carotene desaturase (*ZDS*), and carotenoid isomerase (*CRTISO*), and then lycopene is cyclized into α-carotene and β-carotene by lycopene ε-cyclase (*LCYE*) and lycopene β-cyclase (*LCYB*) [24,25,26]. At this point, the pathway divides into two branches. One is the α-branch, which is the one that transforms α-carotene to lutein in the presence of enzymes encoding cytochrome P450-type ε-ring hydroxylase (*LUT1*) and β-ring hydroxylase (*LUT5*) [27]. An additional branch is the β-branch, which is the conversion of β-carotene to zeaxanthin in the presence of two nonheme iron-dependent β-carotene hydroxylases (*BCH1*, *BCH2*) [28]. The biosynthesis of zeaxanthin and lutein completes the basic carotenoid biosynthesis pathway in these two branches.

The primary ways in which carotenoid biosynthesis is regulated are through the transcriptional levels of the genes and enzymes involved in the process, then through the control of the kind and concentration of carotenoids [29]. Essential enzymes in the carotenoid metabolic pathway, such as *LUT1*, *LUT5*, and *BCH2* genes, have been identified, thoroughly examined, and utilized in the genetic manipulation of carotenoid metabolism and anabolism in other plants, including *Arabidopsis thaliana* [30], *Citrus sinensis* [31], and have been well-studied and used to the genetic engineering of carotenoid synthesis and metabolism. *LUT1* and *LUT5* are the most typical ferredoxin-dependent and nonheme-type enzymes with cyclic specificity in higher plants [32] and are necessary for lutein to be produced from α-carotene and zeaxanthin [33]. Mutations in the *LUT1* locus in *A. thaliana* have been reported to reduce lutein content by 80–95% and lead to the accumulation of zeaxanthin [30]. Overexpression of *AtLUT5* in orange carrots (*Daucus carota*) reduced total carotenoids in roots by 30% to 50% and greatly reduced α-carotene levels [34]. Furthermore, research has shown that upregulating both *VrLUT1* and *VrLUT5* concurrently causes mung beans’ (*Vigna radiata*) lutein and α-carotene contents to rise and fall, respectively [35]. *BCH2* affects zeaxanthin biosynthesis [36]. Overexpression of *CsBCH2* in calli of citrus (*s*) can increase the proportion of lutein and regulate carotenoid biosynthesis [31]. In summary, in the carotenoid metabolic pathway, *LUT1*, *LUT5*, and *BCH2* genes are intimately associated with lutein and zeaxanthin biosynthesis.

Based on the investigation of the ornamental characteristics of *Impatiens* plants in the flower market and our group, we found that *I. hawkeri* ‘Sakimp005’ has a unique color change in the leaves during the growth and development process. At present, there are fewer studies on the color of the leaves of *Impatiens* plants both domestic and foreign, so *I. hawkeri* ‘Sakimp005’ has a very important research value in the study of leaf color change. Therefore, we used a combination of techniques, such as determination of chromaticity value and pigment content, integrative analysis of metabolomics and transcriptomics, and validation of qRT-PCR at the gene expression level, to explore the causes of leaf coloring. This is of great significance in guiding the directional cultivation of *Imaptiens* plants and will also provide important technical support for the study of molecular regulatory mechanisms of color-leaf traits in herbaceous plants.

## 2. Result

### 2.1. Measurement of Colorimetric Values

The brightness L*, red-green parameter a*, yellow-blue parameter b*, and chroma c* values in *I. hawkeri* ‘Sakimp005’ were shown to have a substantial positive correlation with the color change in the leaves during four developmental stages, as indicated in Table 1. In comparison to S2-C, S3-C, and S4-C, the L*, a*, b*, and c* values of the pure green leaves in S1-G were much lower. There was a notable increase in the L*, a*, b*, and c* values in the S2-C and S3-C compared to the S1-G. The leaves began to appear yellow in the S2-C stage, and the yellow coloration of the leaves in the S3-C gradually deepened to a characteristic bright yellow color. During the S4-C stage, leaves turned whitish-yellow, with maximum values for L* and a* and a considerable drop for b* and c*.

### 2.2. Determination of the Pigment Content

**Figure 1:**
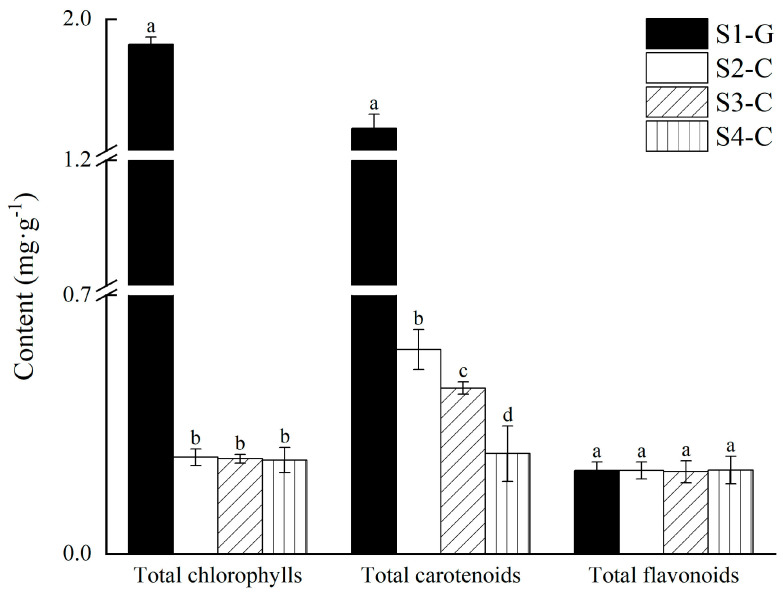
Determination of total chlorophylls, total carotenoids, and total flavonoids in the leaves of *I. hawkeri* ‘Sakimp005’ at four developmental stages. Different lowercase letters indicate significant differences (*p* < 0.05).

The stage of S1-G pure green leaves in Figure 1 had the maximum chlorophyll concentration at 1.905 mg·g^−1^; after the leaves reached S2-C, there was a sharp decrease in chlorophyll content; the leaves in the other three developmental stages (S2-C, S3-C, and S4-C) displayed varying degrees of the yellowing phenomenon. Nonetheless, there was no discernible variation in the three phases’ chlorophyll contents from one another, indicating that chlorophyll might not be the main factor causing *I. hawkeri* ‘Sakimp005’ leaves to appear yellow. Furthermore, since the total flavonoid content was lower than chlorophylls and carotenoids in the four developmental stages (S1-G, S2-C, S3-C, and S4-C) and there was no noteworthy difference, it was clear that flavonoids were not the main cause of the yellow coloration of the leaves of *I. hawkeri* ‘Sakimp005’. When the leaves did not exhibit yellowing, the S1-G had the maximum carotenoid concentration, measuring 1.585 mg·g^−1^. However, at the time of yellowing, the carotenoid content of the S2-C, S3-C, and S4-C dropped to 0.553 mg·g^−1^, 0.448 mg·g^−1^, and 0.271 mg·g^−1^, appropriately. Carotenoids were found to exhibit a consistent decline in four distinct developing stages in the leaves of *I. hawkeri* ‘Sakimp005’, and there was a significant difference between the two comparisons of the four developmental stages. Thus, we hypothesized that at three distinct developmental stages (S2-C, S3-C, and S4-C), variations in the carotenoid concentration may have contributed to the yellowing of the central portion of the leaves in *I. hawkeri* ‘Sakimp005’.

### 2.3. Metabolomic Analysis

Based on colorimetric values and pigment content measurements, we deduced that carotenoids are crucial for the coloration of leaves in *I. hawkeri* ‘Sakimp005’ at four different stages of development. Consequently, we carried out a metabolomics analysis focused on carotenoid targets.

**Figure 2:**
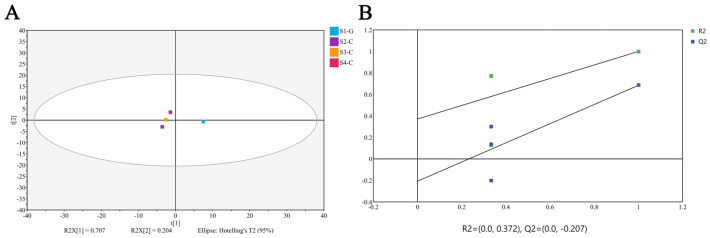
PLA-DA assay. (**A**) PLS-DA score diagram; (**B**) PLS-DA replacement inspection diagram.

**Figure 3:**
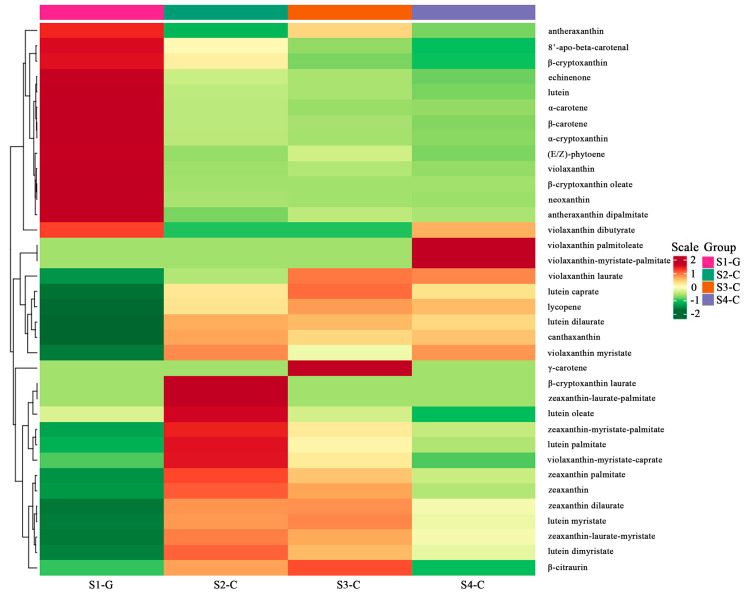
Clustering heatmap of various carotenoids in the leaves of *I. hawkeri* ‘Sakimp005’ at four developmental stages. Red indicates a high level and green indicates a low level.

All samples during metabolomics analysis showed strong peak presentation, retention time reproducibility, and total ion currents (Figure S2). Using internal standards for data quality control and normalization to eliminate redundancy, 68 carotenoid metabolites were examined in four groups of samples, including 61 luteins and 7 carotenoids, and 36 carotenoid metabolites were finally identified in the four groups of samples; only α-carotene, β-carotene, (E/Z)-phytoene, γ-carotene, and lycopene belong to the carotenoid macro-class, while the remaining 31 species belong to the lutein macro-class. Testing the stability of the entire analytical procedure as well as the general distribution among the samples was accomplished using PLS-DA, and the results indicate significant metabolic differences and segregation among the four sample groups (Figure 2A). Figure 2B displays the replacement test for the PLS-DA model. Furthermore, the four sets of samples showed varying amounts of carotenoid metabolites from various species (Figure 3).

**Figure 4:**
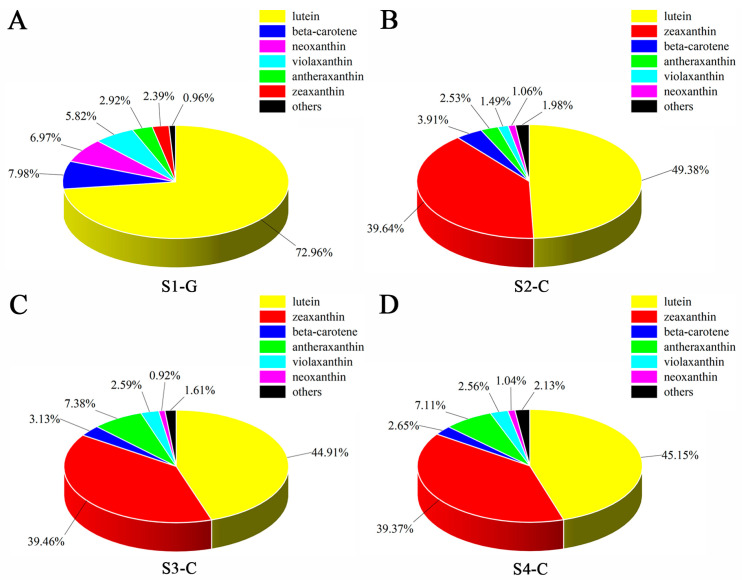
Proportion of different carotenoids in the leaves of *I. hawkeri* ‘Sakimp005’ at four developmental stages. (**A**) S1-G; (**B**) S2-C; (**C**) S3-C; and (**D**) S4-C.

**Figure 5:**
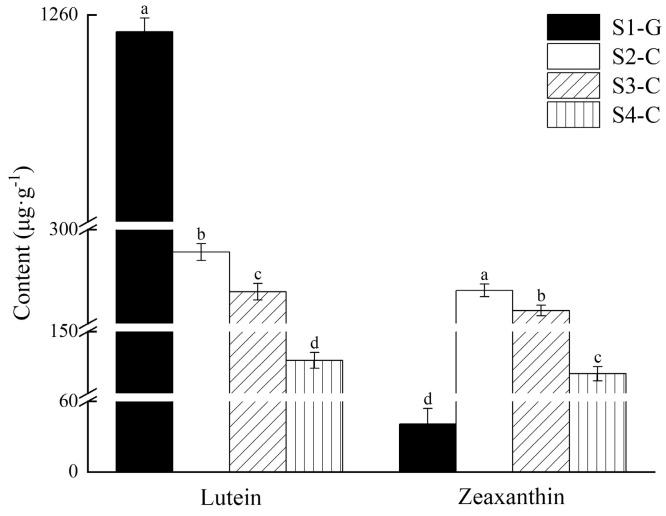
Lutein and zeaxanthin content in the leaves of *I. hawkeri* ‘Sakimp005’ at four developmental stages. Different lowercase letters indicate significant differences (*p* < 0.05).

By employing differential analysis of carotenoid metabolites, a total of 22 carotenoid differential metabolites was identified in the four sets of samples; via mapping the DEMs to the KEGG database, the metabolic pathways were reinforced. Four groups of samples were considerably enriched for metabolites in the carotenoid metabolic pathway, according to enrichment analysis of metabolic pathways based on metabolite up- or down-regulation (Figure S3). Comparing the DEMs of the four sample groups, we found that the pure green leaves in the S1-G had the highest number of DEMs compared to the whitish-yellow leaves in the S4-C (19 in total, with 13 up-regulated and 6 down-regulated); on the contrary, the bright yellow leaves in the S3-C had the lowest number of DEMs compared to the whitish-yellow leaves in the S4-C (2 in total, both down-regulated) (Table S2). Compared with all carotenoid differential metabolites, lutein had the highest percentage in all four stages; zeaxanthin had a smaller percentage in S1-G, but was second only to lutein in S2-C, S3-C, and S4-C (Figure 4). In addition, the changes in lutein content and total carotenoids were consistent with a gradual decrease in the four stages; zeaxanthin content was the lowest in S1-G, and the trend of change was also a gradual decrease in S2-C, S3-C, and S4-C (Figure 5). So, we speculated that it is the two carotenoids (lutein and zeaxanthin), that undergo different changes in composition and percentage in the leaves of *I. hawkeri* ‘Sakimp005’ at four different developmental stages, which result in different degrees of yellowing of the leaves.

### 2.4. Transcriptomic Analysis

#### 2.4.1. Filtering and Analyzing Transcriptome Assembly Data

RNA-seq technology was utilized to sequence the transcriptomics of leaves from four developmental stages to learn more about the process of leaf pigmentation in *I. hawkeri* ‘Sakimp005’. Overall, the sequencing filtering was of acceptable quality and could be used for future transcriptome studies. After removing spliced sequences, ambiguous reads, and low-quality reads, 222,185,234 clean reads were retrieved out of the 226,964,678 raw reads that were generated. In all four groups of samples, Q20 base percentage ranged from 98.30% to 98.33%, Q30 base percentage ranged from 94.76% to 94.85%, and the GC content between 45.67% and 46.93%, with an error rate of 0.02% in each case (Table S3). Utilizing transcriptome assembly, 126,255 transcripts with an average length of 1365 bp and a range of 203 bp to 22,998 bp were found (Figure S4). After assembly, the distribution of unigenes with varying lengths revealed that genes spanning from 200 to 1800 bp made up almost 63% of the total, and the results of splicing indicate that the N50 length was 1668 bp (Table S4).

#### 2.4.2. Functional Annotation Analysis of Genes

**Figure 6:**
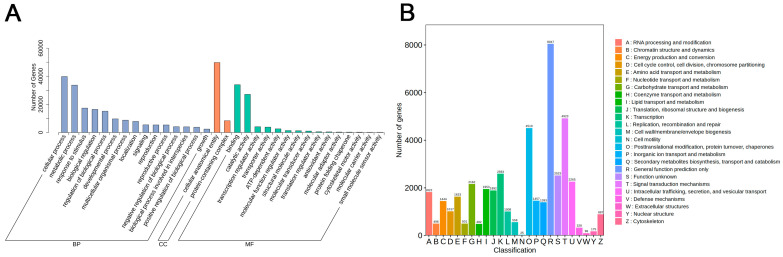
Unigenes function annotation diagrams. (**A**) GO annotation diagram; (**B**) KOG annotation diagram.

We annotated using the KEGG, NR, Swiss-Prot, GO, KOG, and Trembl databases to investigate the function of unigenes (Figure S5). The gene sequence of *I. hawkeri* ‘Sakimp005’ had the highest similarity with *Impatiens glandulifera* among them, with 87.73%, according to the results of the NR database annotation (Figure S6). According to the results of the GO database annotation, unigenes were most commonly annotated as biological processes (BP), then as molecular functions (MF), and finally as cellular components (CC) less frequently. These three major categories of functions were crucial to understanding how *I. hawkeri* ‘Sakimp005’ produces its leaves’ color (Figure 6A). The KOG database annotation results reveal that the categories with the highest number of genes were “General function prediction only”, “Signa transduction mechanisms”, and “‘Posttranslational modification, protein turnover, chaperones’ functions” (8047, 4923, and 4510). The category with the lowest number of genes annotated as cellular activities was “cell turnover”, with only 25 genes (Figure 6B). It followed that there was a close relationship between these functional genes and the color variations in *I. hawkeri* ‘Sakimp005’ leaves.

#### 2.4.3. Analysis of DEGs in Transcriptome Data

**Figure 7:**
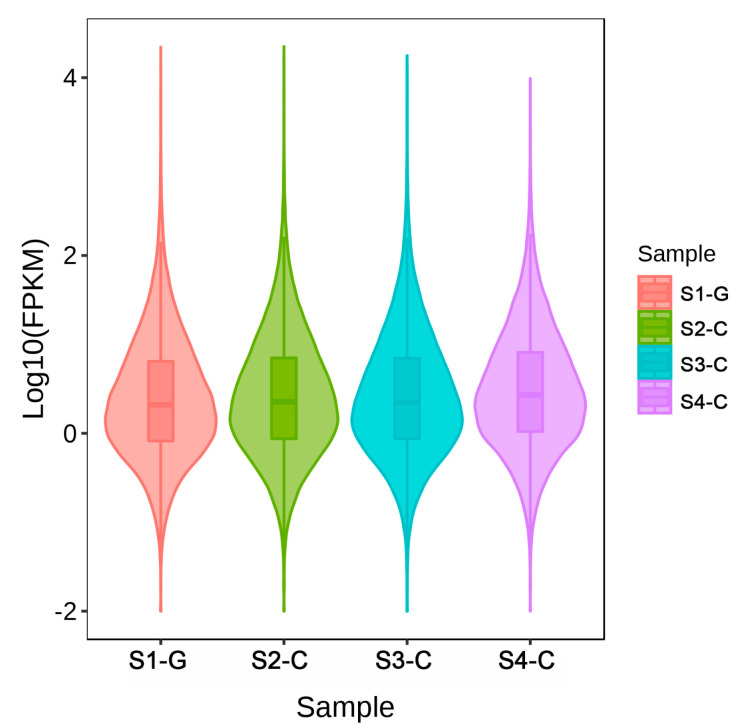
Violin diagram of gene expression.

To ensure the correctness of the subsequent investigations, we first corrected for the sequencing depth and then for the length of the genes or transcripts. For each sample, we normalized the number of mapped reads as well as the transcript length. For further investigation, FPKM was employed as a transcript or gene expression level measure. The distribution showed that the levels of gene expression were consistent throughout the samples, indicating that these findings may be applied to further differential gene analysis (Figure 7). The expression level-based correlation analysis of the samples revealed a strong relationship between the samples in each group, as shown in Figure S7. It was notable that two groups of samples (S2-C and S3-C) had a strong connection with the gene expression of S1-G, even though the gene expression of S4-C had a poor correlation coefficient with the other stages. We suggested that the differential expression of genes in the S4-C stage plays a significant role in the late leaf color changes. The expression patterns of all differently expressed genes (DEGs) in each sample were then investigated using K-mean clustering to investigate the transcriptional differences across samples from different developmental stages (Figure S8). Two clusters were able to be formed from the DEG expression patterns thanks to this study. The first cluster had 9137 DEGs (59% of all DEGs); the average expression of genes in S2-C was lower, whereas the average expression of genes in S3-C and S4-C was greater. Conversely, the second cluster included 7338 DEGs (41% of all DEGs), with a greater average expression of genes in S2-C and a lower average expression of genes in S3-C.

#### 2.4.4. Functional Analysis of DEGs

**Figure 8:**
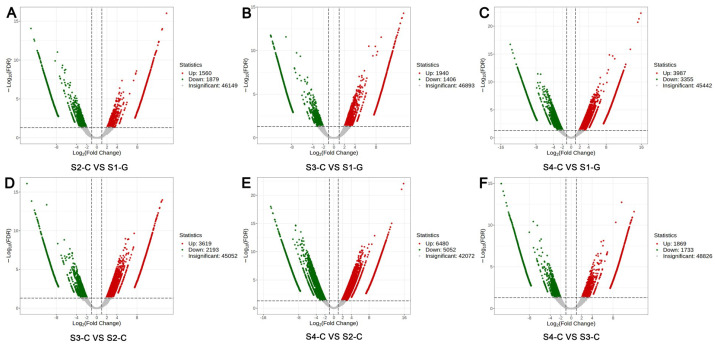
DEG volcano diagrams for each comparison group. (**A**) S2-C vs. S1-G; (**B**) S3-C vs. S1-G; (**C**) S4-C vs. S1-G; (**D**) S3-C vs. S2-C; (**E**) S4-C vs. S2-C; and (**F**) S4-C vs. S3-C.

**Figure 9:**
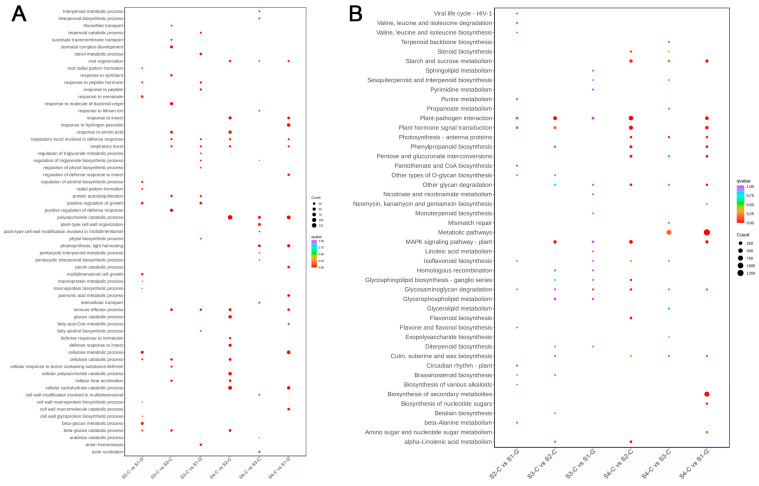
DEG function enrichment bubble maps. (**A**) GO enrichment bubble map; (**B**) KEGG enrichment bubble map.

When the DEGs of the four sample groups were compared, they revealed that S2-C vs. S1-G had 49,588 DEGs (1560 genes up-regulated and 1879 genes down-regulated); S3-C vs. S2-C had 50,864 DEGs (3619 genes up-regulated and 2193 genes down-regulated); S3-C vs. S1-G had 50,239 DEGs (1940 genes up-regulated, 1406 genes down-regulated); S4-C vs. S2-C had 53,604 DEGs (6480 genes up-regulated, 1733 genes down-regulated); S4-C vs. S3-C had 52,428 DEGs (1869 genes up-regulated, 1733 genes down-regulated); and S4-C vs. S1-G had a total of 52,784 DEGs (3987 genes up-regulated, 3355 genes down-regulated); in total, S4-C vs. S2-C had the most DEGs (Figure 8). Subsequent examination of the genes that were differentially expressed in the four samples revealed that the GO enrichment results demonstrate a significant enrichment of DEGs in the cellulose metabolic process, the cellular carbohydrate catabolic process, and the polysaccharide catabolic process (Figure 9A). KEGG enrichment results show that DEGs were mainly enriched in metabolic pathways, biosynthesis of secondary metabolites, plant–pathogen interactions, and other metabolic pathways (Figure 9B). From this, we hypothesized that the coloration of the leaves of *I. hawkeri* ‘Sakimp005’ was closely related to the function and metabolic pathways of DEGs.

### 2.5. Analysis of the Correlation Between DEGs and DEMs

**Figure 10:**
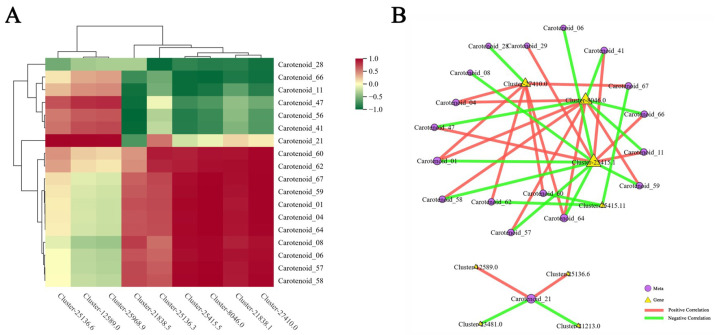
Integrative analysis of DEGs and DEMs. (**A**) Heatmap of correlation clustering between DEMs and DEGs; (**B**) co-expression network analysis diagram of DEMs and DEGs.

We evaluated the correlation between DEGs and DEMs for each of the four sample groups using Pearson correlation coefficients to compute gene expression and metabolite abundance. The metabolites of the four sample groups were lutein, zeaxanthin, β-carotene, octahydroxy lycopene, and antheraxanthin; these were correlated with the *LUT* gene family, the *BCH* gene family, the *LCY* gene family, the *NCED* gene family, and the *NAC* transcription factor family (Figure 10A). The correlation between DEMs and DEGs of the four sample groups was compared and analyzed to arrive at these conclusions. By using a clustering heatmap analysis of DEGs and DEMs, the important DEGs and DEMs were filtered for correlation network analysis. The findings indicate that the DEGs related to these core metabolites were *NCED2* (Cluster-8046.0), *CYP707A* (Cluster-27410.0), *D27* (Cluster-25415.11), and others. The core metabolites were identified as lutein (Carotenoid_59), zeaxanthin (Carotenoid_56), octahedron lycopene (Carotenoid_06), etc. (Figure 10B).

### 2.6. Screening and Analysis of Gene Expression Linked to the Carotenoid Biosynthesis

**Figure 11:**
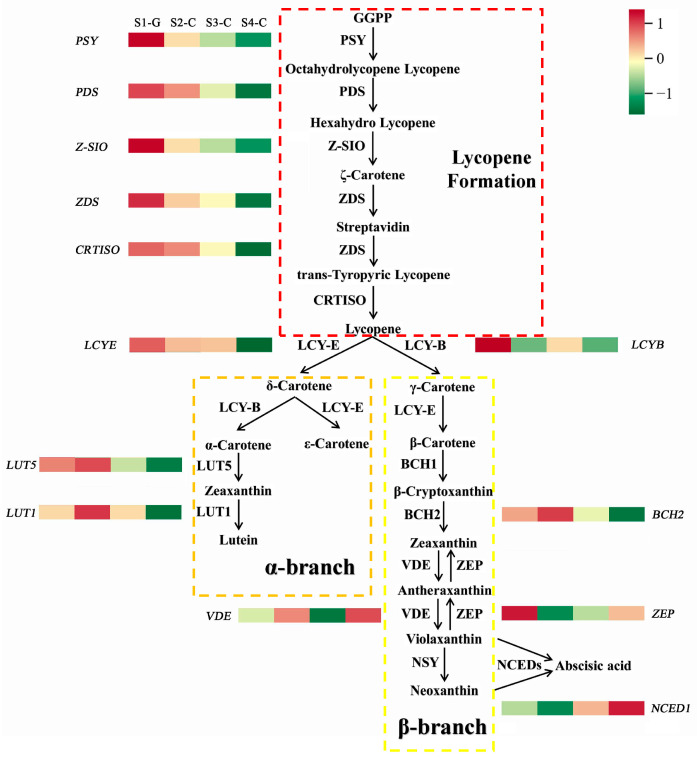
Heatmap of carotenoid metabolic pathways. Red indicates the gene is up-regulated and green indicates the gene is down-regulated.

We discovered that the carotenoid biosynthesis pathway was the primary location for DEGs and DEMs based on the integrative transcriptomics and metabolomics analysis results shown above. As a result, we thoroughly examined the carotenoid metabolic pathway and produced its heatmap (Figure 11).

**Figure 12:**
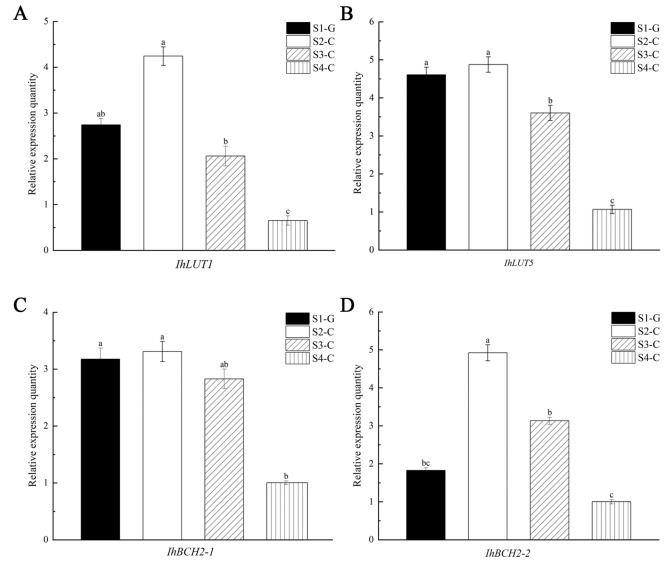
The expression pattern of carotenoid biosynthesis-related genes in the leaves of *I. hawkeri* ‘Sakimp005’ at four developmental stages. (**A**) *IhLUT1*; (**B**) *IhLUT5*; (**C**) *IhBCH2-1*; and (**D**) *IhBCH2-1*. Different lowercase letters indicate significant differences (*p* < 0.05).

We used qRT-PCR to determine the expression level of genes in the leaves of *I. hawkeri* ‘Sakimp005’ at four developmental stages. We accomplished this by analyzing the differences between genes and metabolites in the four groups of samples. We also screened the *IhLUT1*, *IhLUT5*, and *IhBCH2* genes related to lutein and zeaxanthin biosynthesis (Figure 12). The results show that in all four sample groups, the genes were expressed, and that the gene expression patterns agreed with the transcriptomic data, suggesting a high degree of confidence in the transcriptomic data. The *IhLUT1*, *IhLUT5*, *IhBCH2-1*, and *IhBCH2-2* genes all exhibited an increasing and then decreasing trend in expression from the S1-G to S4-C, with the highest expression occurring in the S2-C stage and the lowest in the S4-C stage. The differences in expression between the S1-G and S4-C stages were approximately 6.50, 4.57, 3.30, and 4.91 folds, respectively. We could further speculate that the *IhLUT1*, *IhLUT5*, *IhBCH2-1*, and *IhBCH2-2* genes play an important role in the process of leaf coloration in *I. hawkeri* ‘Sakimp005’. The rationale for this was that the patterns of lutein and zeaxanthin contents aligned with the expression patterns of the four genes during the four distinct stages of leaf growth.

## 3. Discussion

### 3.1. Explanation of Leaf Coloration in I. hawkeri ‘Sakimp005’ Based on Plant Phenotypic Observations and Physiological and Biochemical Indicators

In the study of ornamental plants, CIELAB color space can be used to calculate and compare the brightness, chromaticity, hue, and color metrics of plant leaves at different developmental stages or under different conditions. In addition, some studies used CIELAB measurements to link physiological changes in plants with visual characteristics to accurately measure leaf color changes at different developmental stages [37,38]. We were able to observe the differences in leaf color changes between the four developmental stages of *I. hawkeri* ‘Sakimp005’ in this study. S1-G was green, whereas S2-C, S3-C, and S4-C were yellow, bright yellow, and whitish-yellow, respectively. The L* and a* values of S2-C, S3-C, and S4-C were higher than those of S1-G, and the chromaticity values were consistent with the plant leaf phenotypes that were observed. The findings from earlier research are in line with this [39]. As a result, we deduced that the chromaticity values and color variations across leaves at various phases were in line with plant phenotypic evidence.

A significant group of secondary metabolites known as flavonoids is responsible for the yellowing of leaves and is essential in the regulation of cellular physiology, signaling, and the connections between the plant and its surroundings [40]. The primary component of green plants, chlorophyll, is one of the most significant pigments in photosynthesis; it is responsible for taking in light energy and transforming it into energy, which is how leaves obtain their green color [41]. One type of pigment in photosynthetic processes in plants is called carotenoids. Carotenoids naturally trap light energy and send it to the chloroplasts, where it is used to protect the plant from oxidative damage [42,43]. Generally speaking, variations in the amounts and proportions of carotenoids, flavonoids, and chlorophyll have a direct impact on the color of plant leaves [44]. In our study, chlorophylls were much higher in S1-G than in S2-C, S3-C, and S4-C, but there was no significant difference in the subsequent three. This was similar to previous findings that green leaves have increased chlorophyll content compared to yellow leaves [12]. Flavonoids were the lowest and did not change significantly between leaves at different developmental stages. Although there was a significant decreasing trend in carotenoid content at the S1-G, S2-C, S3-C, and S4-C stages, the content was higher than that of total flavonoids and chlorophylls, so we hypothesized that carotenoids were the main cause of leaf pigmentation in *I. hawkeri* ‘Sakimp005’. Interestingly enough, the S1-G stage’s carotenoid content was much higher than it was in the latter three stages. This was in agreement with previous results showing that the carotenoid content of *U. pumila* is higher than that of *U. pumila* ‘Jinye’ [21]. However, S1-G did not exhibit yellowing, which we surmised was because the leaves’ green color was caused by a higher concentration of chlorophyll than carotenoids at that stage. On the other hand, in the S2-C to S4-C stages, the content of carotenoids decreased but was higher than that of chlorophyll, resulting in the yellow color of the leaves. As integral components of the photosynthetic apparatus, carotenoids are synthesized in a coordinated manner with chlorophyll biosynthesis in chloroplasts, and defects in chlorophyll biosynthesis and chloroplast formation usually result in reduced carotenoid biosynthesis or levels [45]. The middle portion of the leaf started to yellow between stages S1-G and S2-C. At this point, the chlorophyll concentration dropped significantly, possibly as a result of the middle portion of the leaves’ chloroplasts breaking during stage S2-C, which disrupted the pathway for the manufacture of chlorophyll. We will further investigate the chlorophyll fluorescence index and chloroplast ultrastructure of the leaves of *I. hawkeri* ‘Sakimp005’, and deeply study the interaction between chlorophyll and leaf color change.

### 3.2. Interpretation of Leaf Color Mechanism of I. hawkeri ‘Sakimp005’ Based on Targeted Metabolomics and Transcriptomics Data

Understanding plant metabolites plays an important role in metabolite identification, molecular breeding and the mechanisms underlying leaf coloration [13,46]. In metabolomic analysis of the upper leaves of sunflower hybrid HY and green CK, DEMs are enriched in the carotenoid biosynthesis pathway [47]. The carotenoid biosynthesis metabolism route was shown to be considerably enriched in our study, which identified a total of 22 DEMs by differential metabolite pathway enrichment analysis. We discovered that the overall carotenoid content progressively dropped in the leaves of the four developmental stages, with lutein serving as the primary carotenoid in stages S1-G and zeaxanthin as the primary carotenoids in stages S2-C to S4-C. We postulated that the difference in lutein and zeaxanthin content and proportion was the cause of the color change observed in the four stages of *I. hawkeri* ‘Sakimp005’ leaves. The trend of lutein content change was similar to that of total carotenoid content change, while zeaxanthin content change showed a tendency to increase and then decrease. Plant transcriptomics analysis is commonly utilized to discover important genes that are differently expressed at different developmental stages because it gives information about quantitative changes in gene expression [12,48,49]. The identification of several important candidate genes using functional annotation and enrichment analysis of DEGs contributed to the understanding of the molecular process underlying leaf coloration [50]. In a study of mutants (yellowing, reddening, and whitening) of *Anthurium andraeanum* ‘Sonate’, Yang et al. found that leaf color abnormalities in the mutants were caused by altered patterns of expression of pigment biosynthesis genes [51]. DEGs from leaves in four stages of the study were enriched in several plant development metabolic pathways, mainly metabolic pathways, biosynthesis of secondary metabolites, and plant–pathogen interactions (Figure 10B). DEGs were more prevalent in the S2-C stage as opposed to the S4-C stage, or the yellow as opposed to the white-yellow stage, according to transcriptome analysis. To better understand the process behind plant leaf colors, metabolomics and transcriptomics integrative data are extremely important. By combining DEMs and DEGs, we found that leaf color of *I. hawkeri* ‘Sakimp005’ is closely related to genes in the carotenoid biosynthesis pathway.

Studies that are pertinent to this topic demonstrated a close relationship between the biosynthesis and metabolism of carotenoids and the color change in plant leaves, and by overexpressing or knocking out genes in the carotenoid biosynthesis pathway, it is possible to change the leaf color of plants and increase the use of colorful foliage plants in gardens [52,53]. Otani et al. [11] modified the carotenoid synthesis pathway in *Ipomoea obscura* and found that the plant’s leaf color changed. The carotenoid content of *Heliopsis helianthoides* ‘HY’ declined, the leaves became lighter in color, mottled colors appeared, and the ornamental period was greatly extended, according to Qin et al. [47]. It has been established that the major carotenoids in elm, celery, and carrot leaves are lutein and zeaxanthin [54,55,56]. According to Yan et al. [57], there was an up-regulation of *LUT1* gene expression as the papaya rind became yellow during the ripening process from green to green. This was consistent with our study’s findings, which showed that when the color of the leaves turned from green to yellow, the expression level of the *IhLUT1* gene in *I. hawkeri* ‘Sakimp005’ was up-regulated, reaching its peak during the S2-C stage. The decrease in total carotenoids caused by the sharp decline in lutein content led us to hypothesize that the α-branch of the carotenoid biosynthesis pathway was enhanced to synthesize more lutein during S2-C. We also hypothesized that the β-branch was suppressed and the expression level of *IhZEP* and *IhNCED1* genes was significantly down-regulated at this time. Although lutein declined substantially in S2-C, the content and percentage of lutein remained highest and stable in S2-C, S3-C, and S4-C, which we attributed to the lutein, as an end-product in the α-branch could not be cleaved, whereas with cleavage of many carotenoids in the β-branch to produce strigolactone (SL) and ABAs, the content and percentage of lutein remained the highest. Total carotenoids and their derivatives were found in higher concentrations in the green leaves of red maple (*A. rubrum*) than in the yellow and red leaves due to the high expression of the *ArLUT5* gene in those leaves [58], which is inconsistent with the results of our study. The study’s findings reveal that *I. hawkeri* ‘Sakimp005’ leaves had the highest level of *IhLUT5* gene expression in the S2-C, when the leaves began to yellow. They also show that lutein and total carotenoids were less abundant in this stage than they were in the S1-G stage, but zeaxanthin was more abundant. We postulated that the *IhLUT1* and *IhLUT5* genes were up-regulated in the leaves of *I. hawkeri* ‘Sakimp005’ to increase the carotenoid content in response to the significant decrease in carotenoid content during S2-C. This resulted in a significant increase in the content of zeaxanthin and the upstream metabolite of lutein during S2-C. It has been observed in earlier research that in unfavorable circumstances, the carotenoid synthesis pathway switches from the α-branch to the β-branch [59]. From the S3-C to the S4-C, there was a steady down-regulation of *IhLUT5* and *IhLUT1* expression, which was much lower than that in the S1-G. We hypothesized that plants shifted the carotenoid biosynthesis pathway from the α-branch to the β-branch in response to the defects caused by the decline in carotenoids and lutein. *BCH* is one of the key rate-limiting enzymes in the carotenoid metabolic pathway that regulates the biosynthesis of zeaxanthin [60]. We found two *IhBCH* genes in the leaves of *I. hawkeri* ‘Sakimp005’, and the *IhBCH1* gene was able to convert β-carotene to β-cryptoxanthin, and the *IhBCH2* gene converted β-cryptoxanthin to zeaxanthin. Wang et al.’s discovery [49] that down-regulating the *BCH2* gene in *Oncidium hybrid orchids* can cause the flowers to turn from brilliant yellow to pale yellow or white yellow in hue is consistent with what we found. In each of the three stages, the degree of yellow coloration on *I. hawkeri* ‘Sakimp005’ leaves decreased to whitish-yellow and the expression level of *IhBCH2* genes was steadily down-regulated. The hypothesis suggested that the high concentration of zeaxanthin that accumulated during the S2-C was the cause of the down-regulation of the *IhBCH2* expression level. This, in turn, caused the β-branch to exhibit up-regulation of *IhNCED1* genes and down-regulation of *IhBCH2*, which was followed by a decrease in the amount of zeaxanthin.

**Figure 13:**
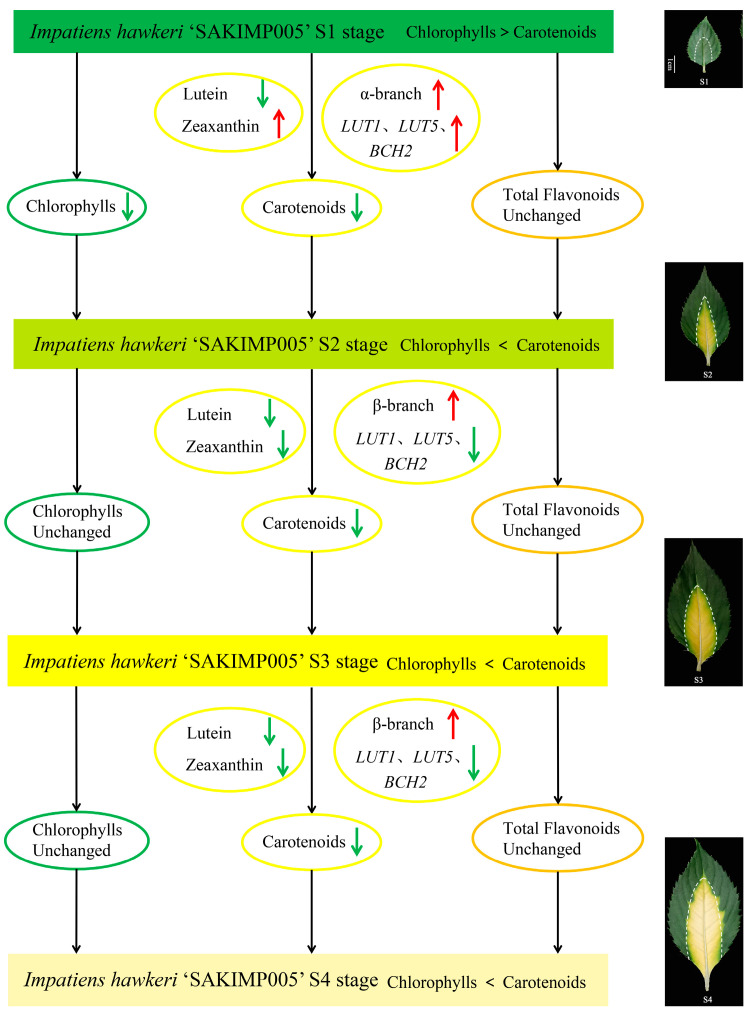
A proposed model for the leaf coloration in *I. hawkeri* ‘Sakimp005’. Red arrows indicate up-regulation and green arrows indicate down-regulation.

We suggested a model for *I. hawkeri* ‘Sakimp005’ leaf coloration based on the findings of our investigation (Figure 13). Among the four leaf developmental stages, S1-G was characterized by green coloration due to higher chlorophyll content than carotenoids, while the S2-C to S4-C stages were characterized by higher carotenoid content than chlorophyll, resulting in yellowing of the leaves. During S2-C, lutein content decreased sharply, the α-branch of carotenoids was enhanced, zeaxanthin content was the highest, and the *IhLUT1*, *IhLUT5*, and *IhBCH2* genes were up-regulated when the yellow color of the leaves began to appear. While in the stages from S3-C to S4-C, the lutein content was still decreasing, which led to the enhancement of the β-branch, decrease in zeaxanthin content, and down-regulation, which further led to the gradual deterioration of the leaves from bright yellow color to whitish-yellow color. Leaf color change in plants is a very complex mechanism involving the biosynthesis of multiple metabolites. Since our study only measured the targeted metabolomics of carotenoids, we did not investigate the effects of other metabolites (e.g., chlorophyll) on leaf coloration, and we did not investigate the changes in chloroplasts during leaf color change in *I. hawkeri* ‘Sakimp005’. In the future, we will further investigate the effects of other metabolites on leaf coloration, as well as determine the chlorophyll fluorescence index, observe the microstructure of chloroplasts, and use transgenic technology to validate the functions of *IhLUT1*, *IhLUT5*, and *IhBCH2* genes, and study the upstream related transcription factors so that the study of the coloration mechanism of *I. hawkeri* ‘Sakimp005’ leaves will be further improved.

## 4. Materials and Methods

### 4.1. Plant Materials

**Figure 14:**
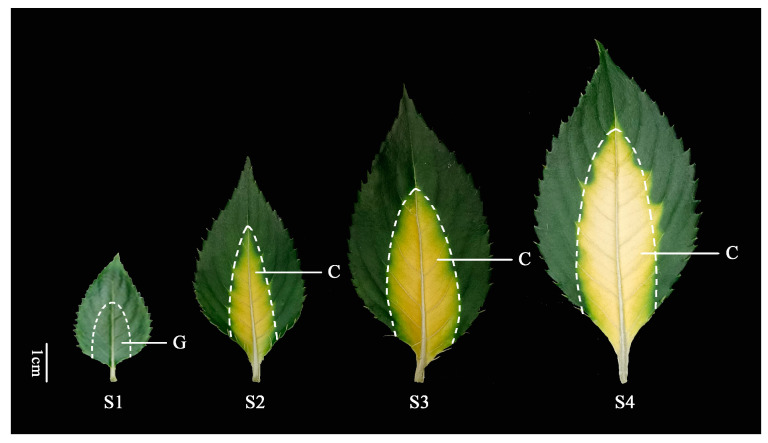
Leaf coloration process in *I. hawkeri* ‘Sakimp005’. S1-S4 were the four crucial developmental stages during leaf coloration; G: green; and C: color.

The material used in this investigation was *I. hawkeri* ‘Sakimp005’ grown in Southwest Forestry University’s arboretum. Growth conditions were maintained at 18–25 °C, with 11–13 h of daylight and 40–60% humidity. Four different developmental stages were classified according to leaf color changes: stage 1 (S1-G) for leaves that were small and pure green; stage 2 (S2-C) for leaves that showed yellow around the main veins but still had green leaf margins, stage 3 (S3-C) for leaves where the yellow portion turned bright yellow, and stage 4 (S4-C) for leaves where the bright yellow color changed to a whitish yellow (Figure 14). Five leaves were taken from each of the ten *I. hawkeri* ‘Sakimp005’ plants that were pest and disease free; the section around the main veins was separated from the plant and rapidly placed in liquid nitrogen. Leaves from all four developmental phases were combined separately, tagged, and refrigerated at −70 °C. Fresh leaves were used to calculate pigmentation and colorimetric values.

### 4.2. Measurement of Colorimetric Values

The average of three measurements was performed after fresh leaves were gathered and measured using the CIE standard D65 light source and CIE standard 10° color function. After the measurements were completed, the International Commission on Illumination’s (CIELAB) color model was used to examine the chromaticity values of the leaf hues [61]. In this case, luminance is represented by L* (which ranges from 0 to 100), chroma is represented by c*, which is (a*^2^ + b*^2^)^1/2^, red and green are, respectively, represented by a* (which positive and negative values), and yellow and blue are, respectively, represented by b* (which positive and negative values) [44,62].

### 4.3. Determination of the Pigment Content

Three biological replicates were carried out for each developmental stage to determine the chlorophyll and carotenoid contents by Porra [63]. The following computation formulas were used:

Ca (mg·L^−1^) = 12.7*OD_663_ − 2.69*OD_645_,

Cb (mg·L^−1^) = 22.9*OD_645_ − 4.68*OD_663_,

chlorophyll content (mg·g^−1^) = (8.02*OD_663_ + 20.21*OD_645_)*V/1000/m,

and carotenoid content (mg·g^−1^) = (4.367*OD_470_ − 0.014*Ca − 0.454*Cb)*V/1000/m.

V represents the extract’s volume (mL), while m stands for the leaves fresh weight (g). The extracts’ absorbance values at 470 nm, 645 nm, and 663 nm were, respectively, OD_470_, OD_645_, and OD_663_. Chlorophyll a’s mass concentration is represented as Ca (mg·L^−1^), whereas chlorophyll b’s mass concentration is represented as Cb (mg·L^−1^).

The specific method for the determination of total flavonoid content was referred to by Shraim [64]. A standard solution of 0.77 mg·L^−1^ rutin was prepared by adding 6 mL of 1% AlCl_3_-6H_2_O methanol solution, and the absorbance (D270 nm) standard curve was plotted at 415 nm (Figure S1). The total flavonoid content of each sample was obtained by taking 2 mL of leaf extract from each of the four different developmental stages and then calculated according to the standard curve. Three biological replicates were performed for each developmental stage.

### 4.4. Carotenoid-Targeted Metabolomics Assays Analysis

After being freeze-dried and crushed into a powder (30 Hz, 1.5 min), the samples were kept at −80 °C until they were needed. In total, 50 mg of powder was weighed, and 0.5 mL of a mixed solution of n-hexane, acetone, and ethanol (1:1:1, *v*/*v*/*v*) was used for extraction. At room temperature, the extract was vortexed for 20 min. After five minutes at 4 °C and a centrifugation speed of 12,000 rpm, the supernatants were gathered. The preceding procedures were repeated with the same parameters to remove the residue once more. Subsequently, it dried out and was reconstituted in 100 microliters of dichloromethane. A 0.22 μm membrane filter was used to filter the mixture. The UPLC-APCI-MS/MS (UPLC (Milford, MA, USA), ExionLC™ AD (Framingham, MA, USA), https://sciex.com.cn/; MS. Applied Biosystems 6500 Triple Quadrupole (Thermo Fisher Scientific), https://sciex.com.cn/) equipment was utilized for the analysis of the sample extracts.

Partial least squares discriminant analysis (PLS-DA) was used to analyze the total carotenoid content of each sample using Simaca 14.1. Heatmaps accompanied by dendrograms were used to display the samples’ and metabolites’ hierarchical cluster analysis (HCA). R (1.2.1; 2.7.1.1009) package was used to perform HCA. Color spectrum visualization was used to represent the normalized signal strength intensities of metabolites for HCA. Using absolute log_2_ fold change (FC), significantly regulated metabolites were identified between groups. For differential metabolites (DEMs), the screening parameters were fold change ≥ 2 and fold change ≤ 0.5. Annotations were made to the detected metabolites using the KEGG compound database (http://www.kegg.jp/kegg/compound/, (accessed on 7 July 2024)), and the KEGG pathway database (http://www.kegg.jp/kegg/pathway.html, (accessed on 7 July 2024)) was then mapped to the annotated metabolites. According to the *p*-value obtained by the hypergeometric test, the regulatory pathway of significant metabolite enrichment was determined. Following that, metabolite sets enrichment analysis (MSEA) was fed these pathways.

### 4.5. RNA-Seq Sequencing Analysis

One microgram of total RNA was needed for each sample during processing. Using the NEBNext^®^ UltraTM RNA Library Prep Kit for Illumina^®^ (NEB, Ipswich, MA, USA) following the instructions provided by the manufacturer, sequencing libraries were created, and index codes were added to each sample to identify its sequences. The library fragments were purified by selecting cDNA fragments with lengths between 250 and 300 bp by Beckman Coulter’s AMPure XP technology (Beverly, MA, USA). Subsequently, 3 µL of USER Enzyme (NEB, USA) was integrated with adaptor-ligated, size-selected cDNA and incubated at 37 °C for 15 min. This was followed by 5 min at 95 °C to initiate PCR. After that, PCR was conducted using Phusion high-fidelity DNA polymerase, universal PCR primers, and index (X) primer. Ultimately, the PCR products were separated utilizing the AMPure XP system (Brea, CA, USA), and the library quality was assessed using the Agilent Bioanalyzer 2100 system (Santa Clara, CA, USA). As directed by the manufacturer, the index-coded samples were clustered by a cBot Cluster Generation System and the TruSeq PE Cluster Kit v3-cBot-HS (Illumia). Following clustering, the Illumina platform was used to sequence the library preparations, yielding 150 bp paired-end reads.

Fastp 0.23.2 was used to filter the original data, particularly for measurements obtained with adapters. Trinity v2.13.2 (https://github.com/trinityrnaseq/trinityrnaseq, (accessed on 10 July 2024)) and Corset 1.09 were used to assemble the transcriptome and classify pertinent transcripts into “gene” clusters. Use TransDecoder 5.3.0 (https://github.com/TransDecoder/TransDecoder/wiki, (accessed on 12 July, 2024)) to determine potential coding areas in transcript sequences produced by de novo RNA-seq transcript assembly using Trinity. Using HMMER or DIAMOND v2.0.9, gene function was annotated based on the following databases: Nr (NCBI non-redundant protein sequences); Swiss-Prot (a manually annotated and reviewed protein sequence database); Trembl (a variety of new documentation files and the creation of TrEMBL, a computer annotated supplement to SWISS-PROT); Kyoto Encyclopedia of Genes and Genomes (KEGG); Gene Ontology (GO); KOG/COG (COG: Clusters of Orthologous Groups of proteins; KOG: euKaryotic Ortholog Groups); and Protein family (Pfam). After estimating the levels of gene expression using RSEM v1.3.1, each gene’s fragments per kilobase of transcript per million (FPKM) fragments mapped were determined by taking into account the length of the gene. By applying the Benjamin and Hochberg approach, the *p*-value was adjusted, and to express a substantial difference, the corrected *p*-value and |log_2_ fold change| were utilized as the threshold. The requirements for unique gene screenings were |log_2_ fold change| ≥ 1 and false discovery rate (FDR) < 0.05. The hypergeometric test served as the foundation for the enrichment analysis. To do the hypergeometric distribution test, use the GO term for GO and the route unit for KEGG.

### 4.6. Expression and Analysis of IhLUT1, IhLUT5, IhBCH2-1, and IhBCH2-2 Genes

The expression of *IhLUT1*, *IhLUT5*, *IhBCH2-1,* and *IhBCH2-2* in leaf samples from four developmental stages was examined using quantitative real-time PCR (qRT-PCR).

Using an online program (https://www.ncbi.nlm.nih.gov/tools/primer-blast, (accessed on 15 July 2024)) from the National Center for Biotechnology Information (NCBI), we created primers for five genes: *IhActin*, *IhLUT1*, *IhLUT5*, *IhBCH2-1*, and *IhBCH2-2* (Table S1). The *IhActin* gene was identified by screening from the transcriptomic data in this study. The qPCR SYBR Green Master Mix (10 μL), upstream and downstream primers (0.4 μL each), dd H_2_O (8.2 μL), and cDNA template after 10-fold dilution (1 μL) made up the 20 μL qRT-PCR reaction system. Five minutes of predenaturation at 94 °C, 30 s of denaturation at 94 °C, 30 s of annealing at 60 °C, and 20 s of extension at 72 °C, and 40 cycles made up the reaction program. Each sample had three technical duplicates. The 2^−∆∆Ct^ method was applied to evaluate the genes’ relative expression levels. Data were displayed using Origin 2021 and subjected to significance analysis using SPSS 24.0.

## 5. Conclusions

In the study to investigate the mechanism of coloration in the leaves of *I. hawkeri* ‘Sakimp005’, we performed pigment content and chromaticity values, as well as integrative analysis of metabolomics and transcriptomics. The higher chlorophyll content than carotenoid content may be responsible for the green coloration of S1-G leaves. In contrast, the higher carotenoid than chlorophyll and the variation in lutein and zeaxanthin in terms of content and proportions may be responsible for the different yellowing of leaves from S2-C to S4-C. This study helps to understand the leaf coloration mechanism of *I. hawkeri* ‘Sakimp005’, provides a theoretical basis for horticultural workers to orient the cultivation of colorful foliage varieties of *Impatiens* plants, and will also provide important technical support for the application of herbaceous colorful foliage plants.

## Figures and Tables

**Table 1:** Measurement of colorimetric values of leaves of *I. hawkeri* ‘Sakimp005’ at four developmental stages.

Developmental Stages	L*	a*	b*	c*
S1-G	45.33 ± 0.33 d	−16.33 ± 0.33 d	14.33 ± 0.33 d	21.74 ± 0.24 d
S2-C	62.33 ± 0.33 c	−1.67 ± 0.33 c	54.00 ± 0.67 b	53.03 ± 1.52 b
S3-C	74.67 ± 0.33 b	1.33 ± 0.33 b	63.67 ± 0.58 a	63.68 ± 0.67 a
S4-C	93.00 ± 0.33 a	4.67 ± 0.33 a	28.33 ± 0.88 c	28.73 ± 0.87 c

Different lowercase letters indicate significant differences (*p* < 0.05).

## Data Availability

Due to delayed data release (NCBI; SRA accession number PRJNA1176279), but can be obtained from the corresponding author on reasonable request.

## Supplementary Materials

The supporting information can be downloaded at: https://www.mdpi.com/article/10.3390/ijms26010174/s1.
