# Supplementary material for: Integrative Targeted Metabolomics and Transcriptomics Reveal the Mechanism of Leaf Coloration in Impatiens hawkeri ‘Sakimp005’
Source: Int J Mol Sci. 2024 Dec 28;26(1):174. doi: 10.3390/ijms26010174 (PMC11720316; doi:10.3390/ijms26010174)

Supplementary materials:

Table S1. qRT-PCR primers for *IhLUT1*, *IhLUT5*, *IhBCH2-1*, and *IhBCH2-2* genes.

Primers name	Primers Sequences （5'-3'）	Use
QIhActin F	GTTCTGTCCATTCCCATCTGTC	Upstream primer of *QIhActin*
QIhActin R	CCCTGAGGAATCCAGTGAGC	Downstream primer of *QIhActin*
QIhLUT1 F	CGATGCGAAGCTTGAAGACG	Upstream primer of *QIhLUT1*
QIhLUT1 R	CCATGTTCACCGGAGTTCCA	Downstream primer of *QIhLUT1*
QIhLUT5 F	CTGCGGTCCTAACATGGACA	Upstream primer of *QIhLUT5*
QIhLUT5 R	GGTGGTTGTGGGTAAAGCCT	Downstream primer of *QIhLUT5*
QIhBCH2-1 F	AGGATCCAAGCAAGGAAGGC	Upstream primer of *QIhBCH2-1*
QIhBCH2-1 R	AAACAGCCATGACCGCCATA	Downstream primer of *QIhBCH2-1*
QIhBCH2-2 F	CAAAACGCCTTTCTCCGTCC	Upstream primer of *QIhBCH2-2*
QIhBCH2-2 R	AAGCGCTCCAACTGTTTCCT	Downstream primer of *QIhBCH2-2*

Table S2. Number of DEMs in each comparison group.

Group name	All sig diff	Down regulated	Up regulated
S1-Gvs S2-C	17	10	7
S2-C vs S3-C	5	4	1
S3-C vs S1-G	17	11	6
S4-C vs S2-C	13	13	0
S4-C vs S3-C	2	2	0
S4-C vs S1-G	19	13	6

Table S3. Sequencing data statistics table.

Sample	Raw Reads	Clean Reads	Clean Base(G)	Error Rate(%)	Q20(%)	Q30(%)	GC Content(%)
S1-G	60428856	60428856	8.92	0.02	98.33	94.85	46.88
S2-C	51436570	50348490	7.55	0.02	98.32	94.79	46.93
S3-C	60375630	59046840	8.86	0.02	98.33	94.85	46.60
S4-C	54723622	53324782	8.00	0.02	98.30	94.76	45.67

Table S4. Sample splicing results.

Type	Number	Mean Length	N50	N90
Transcript	126255	1365	1938	720
Unigene	75588	1668	2100	902

Figure S1. Rutin standard curve.

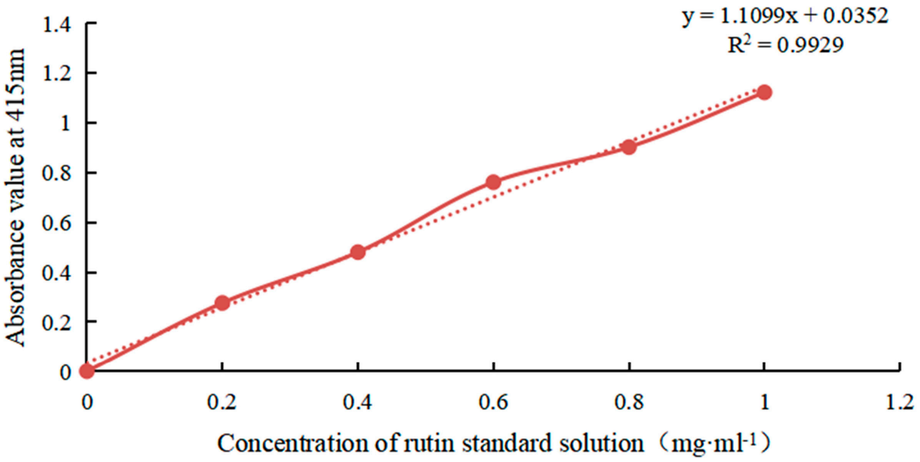

Figure S2. Quality control diagrams for four sample groups.

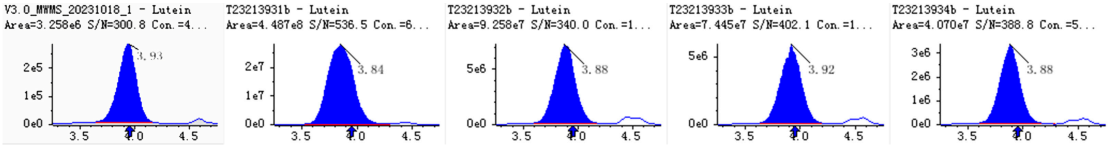

Figure S3. Differential metabolite KEGG enrichment bubble maps. **A:** S2-C vs S1-G; **B:** S3-C vs S1-G; **C:** S4-C vs S1-G; **D:** S3-C vs S2-C; **E:** S4-C vs S2-C; **F:** S4-C vs S3-C.

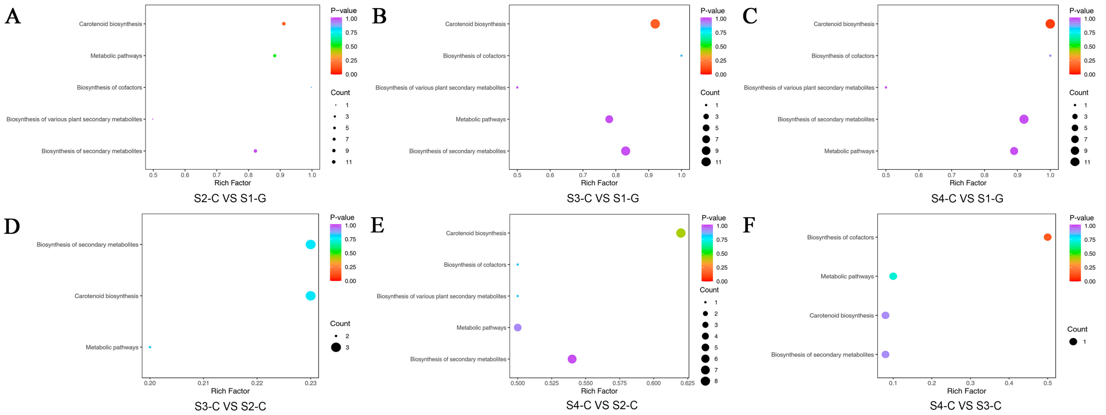

Figure S4. Sequence length distribution diagram.

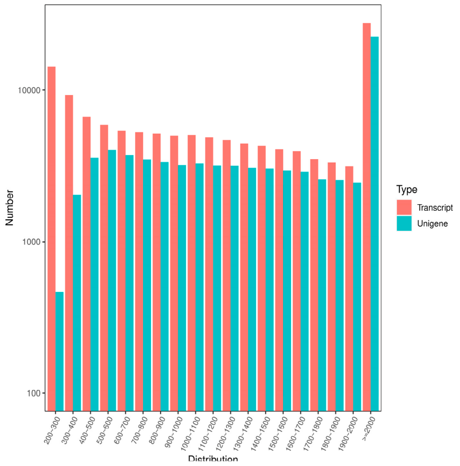

Figure S5. Unigene annotation statistics diagram.

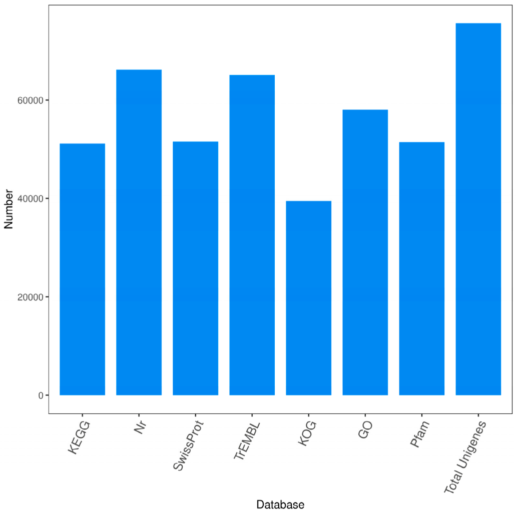

Figure S6. NR annotation diagram.

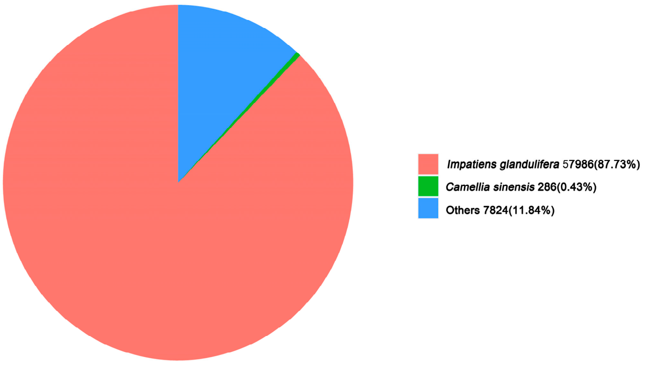

Figure S7. Sample correlation diagram.

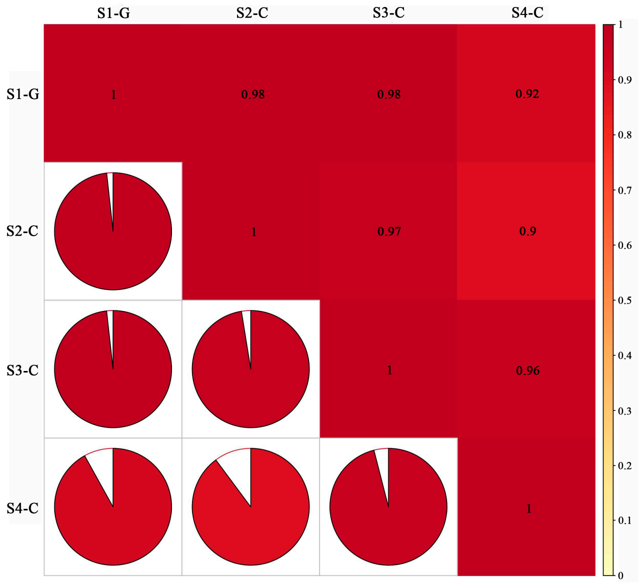

Figure S8. Diagram of K-means clustering results with the specific number of DEGs included in each cluster.

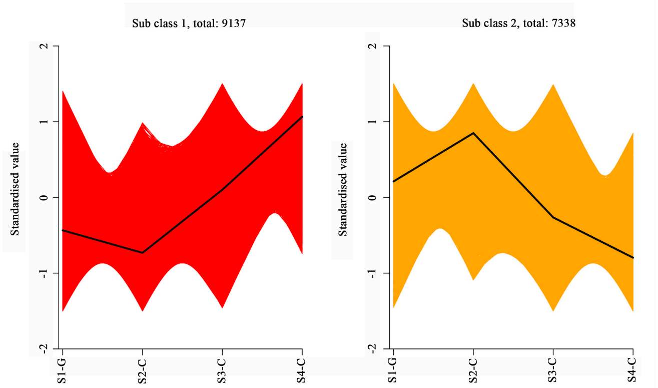

Supplement: Supplementary file 1 [file ijms-26-00174-s001.zip › Supplementary materials.pdf]
